# Supplementary material for: Multiple processes of vocal sensory-motor interaction in primate auditory cortex
Source: Nat Commun. 2024 Apr 10;15:3093. doi: 10.1038/s41467-024-47510-2 (PMC11006904; doi:10.1038/s41467-024-47510-2)
Supplement: Supplementary file 1 — Supplementary Information [file 41467_2024_47510_MOESM1_ESM.pdf]

Supplementary Information for

Multiple processes of vocal sensory-motor interaction in primate auditory cortex

Joji Tsunada, Xiaoqin Wang, Steven J Eliades*

*Correspondence to: steven.eliades@duke.edu

This PDF file includes Supplementary Figures 1-8

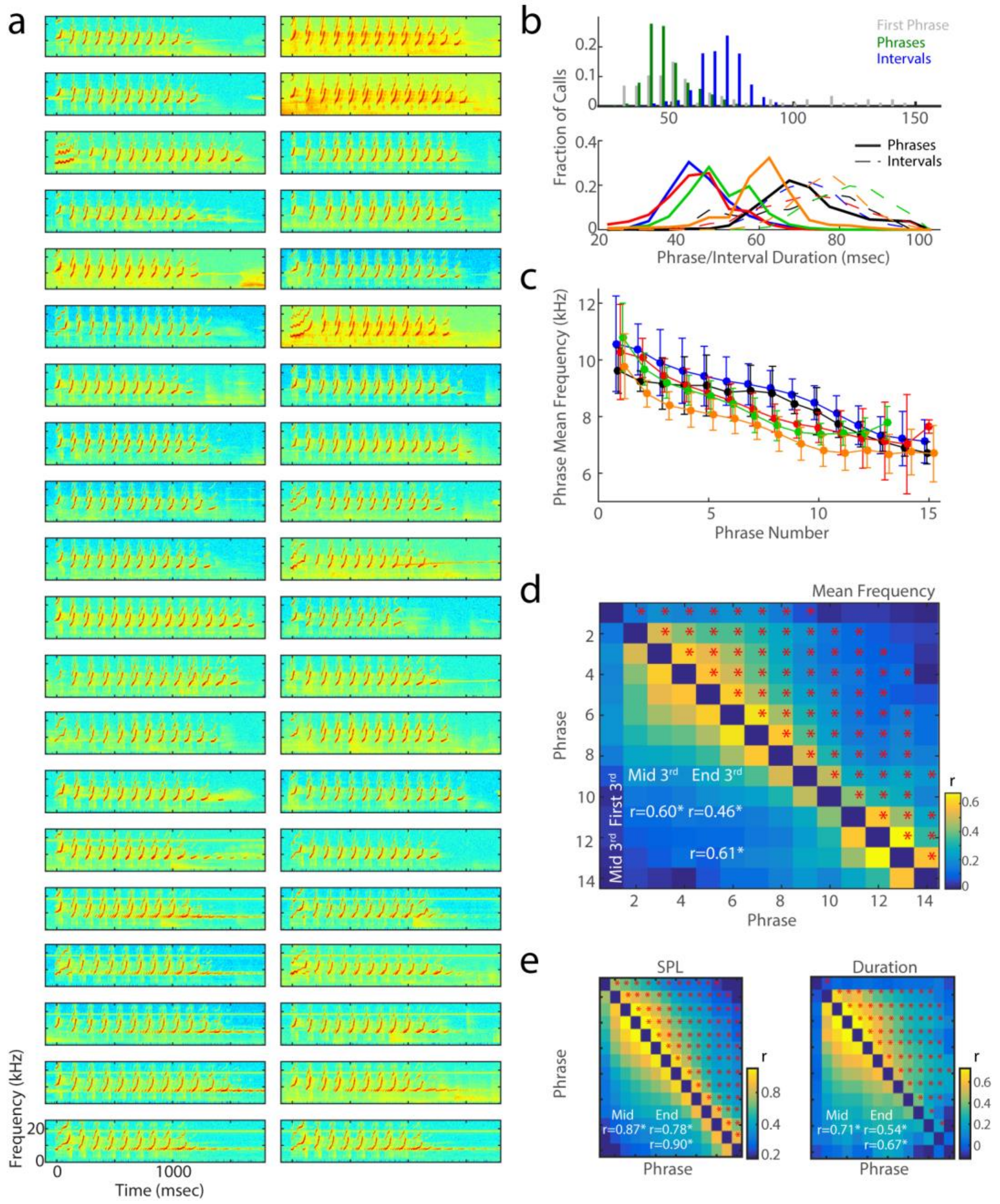

Supplementary Fig. 1: Acoustic analysis of twitter phrases

a Sample of 40 twitters call spectrograms that were part of the unit data shown in Fig. 1. **b** Distributions of twitter phrase and interval durations shown for the unit data in Fig. 1 (top), and for all vocalizations and animals (bottom). First phrase durations were more variable and longer than durations for subsequent phrases and intervals. Acoustics measured for different animals are shown in different colors (bottom). **c** Mean acoustic frequency of twitter phrases averaged for each animal (colors as in **b**), showing a regular progression of frequency between phrases. Mean and standard deviation are shown. **d** Plot showing the correlation coefficients between mean frequency of each twitter phrase. Frequencies were z-scale normalized for each animal and phrase prior to calculating the correlation. Correlations therefore suggest that a given phrase that is above (or below) average for that phrase predicts a similar variation in subsequent phrases. Inset are correlations calculated between the first, middle, and last third of phrases for a given vocalization, normalized as above. (* $p<0.001$, Pearson correlation coefficients with two-sided t-tests, FDR corrected, n=13,804 vocalizations). **e** Correlations plots as in **d**, but for phrase SPL (left) and duration (right), suggesting predictable of subsequent phrase acoustics based upon earlier phrases. Source data are provided as a Source Data file.

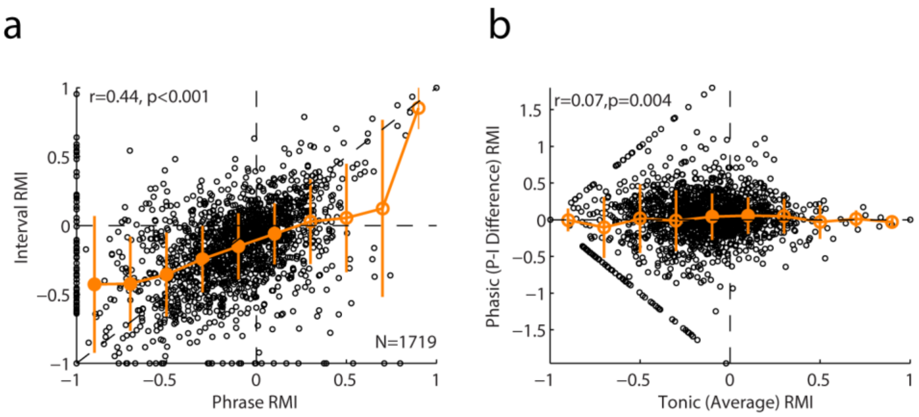

Supplementary Fig. 2: Single-unit twitter responses.

a Scatter plot comparing phrase and interval RMIs, limited to only single-units. Correlation coefficient is indicated (r=0.44, p=$4x10^{-84}$, Pearson correlation with two-sided t-test, n=1719 units). Mean interval RMI binned by phrase response (orange; mean±SEM, filled: p<0.05, two-sided signed-rank tests with FDR corrections, exact p-values in Source Data file) shows a bias towards less interval suppression or excitation for suppressed and excited neurons, similar to that seen for all units (i.e. Fig. 2a). **b** Scatter plot comparing phasic P-I differences against average tonic twitter responses, again limited to single units. Source data are provided as a Source Data file.

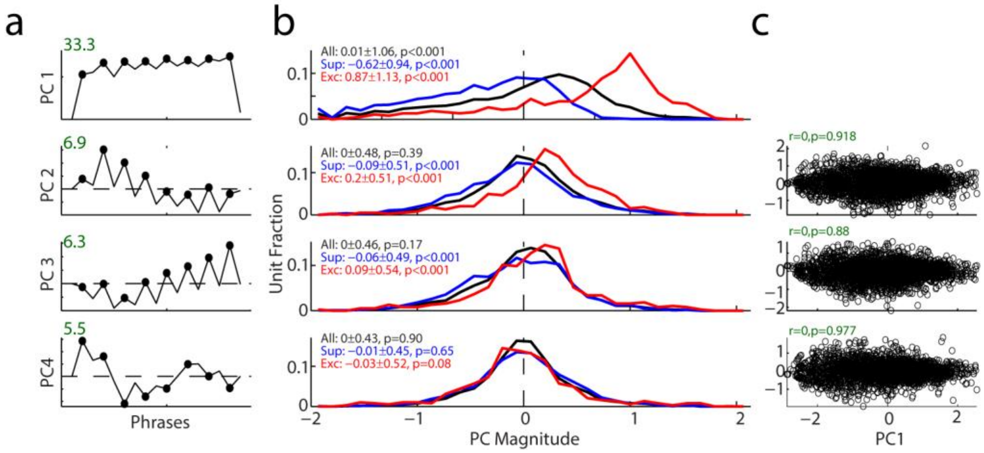

Supplementary Fig. 3: Principal component analysis (PCA) of twitter phrase and interval responses.
a The first four principal components (PCs) are shown, dots indicate phrases. The first PC captured tonic responses across the entire vocalization. The second and third PCs showed phasic responses with phrases greater than intervals, but with descending vs. ascending trends between phrases. The fourth PC appeared to capture a largely onset response. Percent of explained variance for each PC is indicated. **b** Distributions of the four PC projections are shown including mean±s.d. and significance of deviation from zero (PC1: $p=2x10^{-5}$, PC2: p=0.39, PC3: p=0.17, PC4: p=0.90; two-sided signed-rank test, n=3224 units). Overlayed are PC projections sorted for suppressed (RMI≤-0.2, blue) and excited (RMI≥0.1, red) units. Not surprisingly, suppressed units showed a negative tonic PC1 as well as negative phasic PCs 2 and 3 (phasic suppression; PC1: $p=5x10^{-95}$, PC2: $6x10^{10}$, PC3: $1x10^{-4}$, PC4: 0.65; n=1470 units). Excited units showed positive PCs 1-3, consistent with net positive increased response (PC1: $p=x10^{-32}$, PC2: $7x10^{-17}$, PC3: $3x10^{-5}$, PC4: 0.08, n=382 units). **c** Scatter plots comparing PCs 2-4 with PC1, exhibiting absent correlations and consistent with the separability of phasic and tonic vocal responses (Pearson correlations with two-sided t-tests). Source data are provided as a Source Data file.

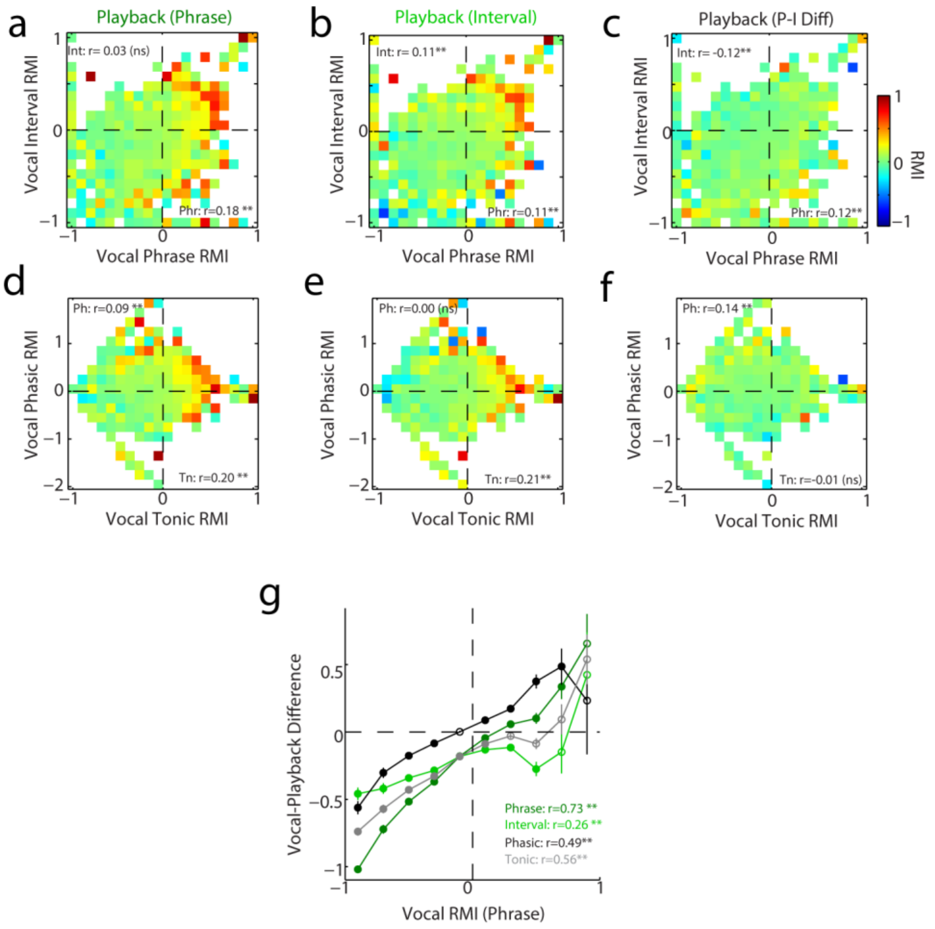

Supplementary Fig. 4: Comparison of vocal production and playback responses.

a,b Playback responses sorted by vocal production phrase/interval (**A**) and phasic/tonic responses (**B**) as in Figure 3. Phrase: Phr, interval: Int, tonic: Tn, phasic: Ph. Partial correlation coefficients are shown (** $p<0.001$; Phr: $p=4\times10^{-25}$, Int: $p=0.06$, Tn: $p=4\times10^{-31}$, Ph: 6×10^{-7}, two-sided t-tests, n=3243 units). **c,d** Sorted responses, but for playback responses during inter-phrase intervals (Phr: $p=9\times10^{-11}$, Int: 2×10^{-10}, Tn: 6×10^{-34}, Ph: 0.83). **e,f** Playback P-I differences showed increased activity during phrases compared to intervals (Phr: $p=2\times10^{-11}$, Int: 7×10^{-12}, Tn 0.65, Ph: 2×10^{-15}). **g** Vocal-playback comparison binned by vocal phrase RMI, shown separately for vocal-playback differences measured during phrases (dark green, identical to Fig. 3e,f), intervals (light green), phasic P-I differences (black), and tonic averages (grey). All showed similar trends with decreased activity during vocalization compared to playback for suppressed units (negative vocal RMI), though inter-phrase intervals showed the weakest correlation. Intervals showed negative differences (decreases during vocal production) even for many excited units (positive vocal RMI), suggesting a tonic inhibitory component. Phrases and phasic components were stronger during vocalization than playback, but only for excited vocal units. Mean±SEM are plotted, filled symbols: $p<0.05$ (two-sided signed-rank tests with FDR corrections, exact p-values in Source Data file), correlations indicated (** $p<0.001$; Phrase: $p<1\times10^{-200}$, Interval: $p=1\times10^{-49}$, Phasic: $p=1\times10^{-199}$, Tonic: $p<1\times10^{-200}$, Pearson correlations with two-sided t-tests, n=3243 units). Source data are provided as a Source Data file.

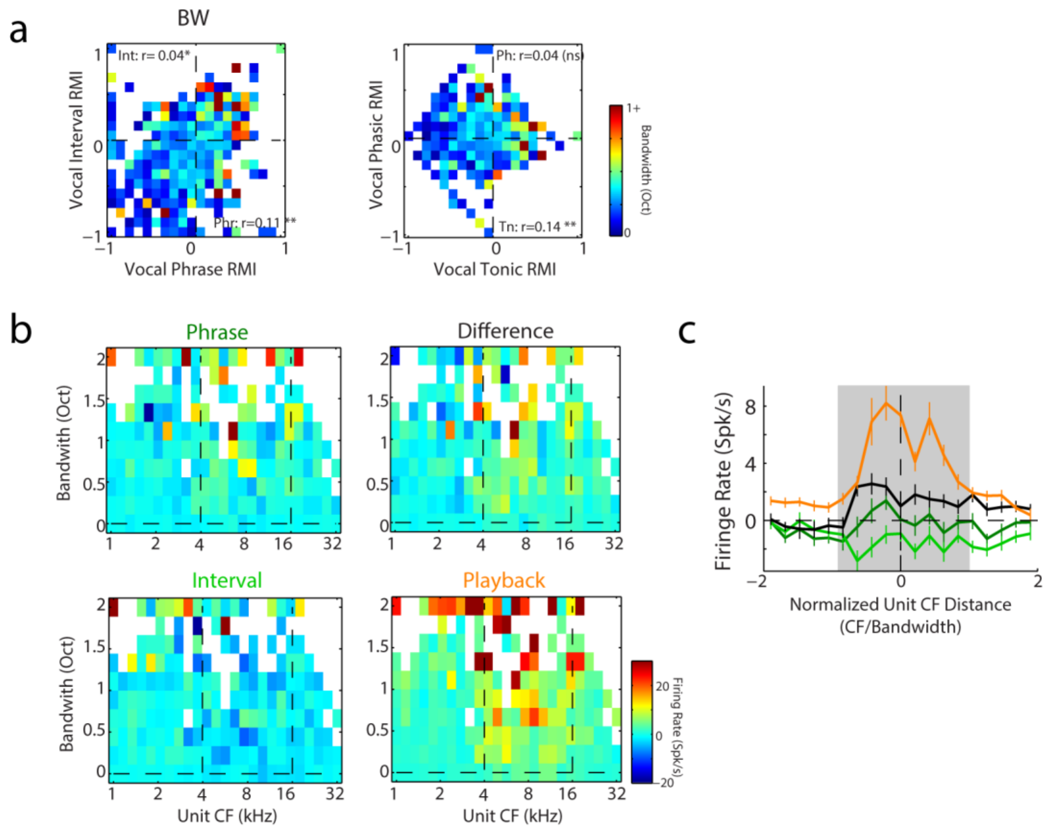

Supplementary Fig. 5: Effects of bandwidth on vocal CF dependence.

a Comparison of frequency tuning bandwidth, in octaves, for units of differing vocal responses sorted by phrase/interval (left) and phasic/tonic (right) responses. Larger bandwidths are seen for units with vocal excitation during phrases/tonic responses. Units with bandwidths >1 have been truncated to 1+. Partial correlation coefficients are indicated (*p<0.05, ** p<0.001, n.s. non-significant; Phr: $p=6x10^{-7}$, Int: p=0.043, Tn: $p=3x10^{-11}$, Ph: 0.097, two-sided t-tests, n=2136 units). **b** Vocal responses during phrases, intervals, phrase-interval differences, and playback are plotted against unit CF and bandwidth. Units with larger bandwidths, or with CF closer to the vocal frequency range (dashed lines) showed stronger responses, particularly during playback where vocal suppression is absent. **c** Firing rate responses are plotted against unit CF distance, as in Fig 4D, but with the distance normalized by unit bandwidth. Mean±SEM are shown, shaded: ±1 octave (n=2136 units). Units with smaller normalized CF distances showed stronger vocal suppression/excitation that units more distant. Source data are provided as a Source Data file.

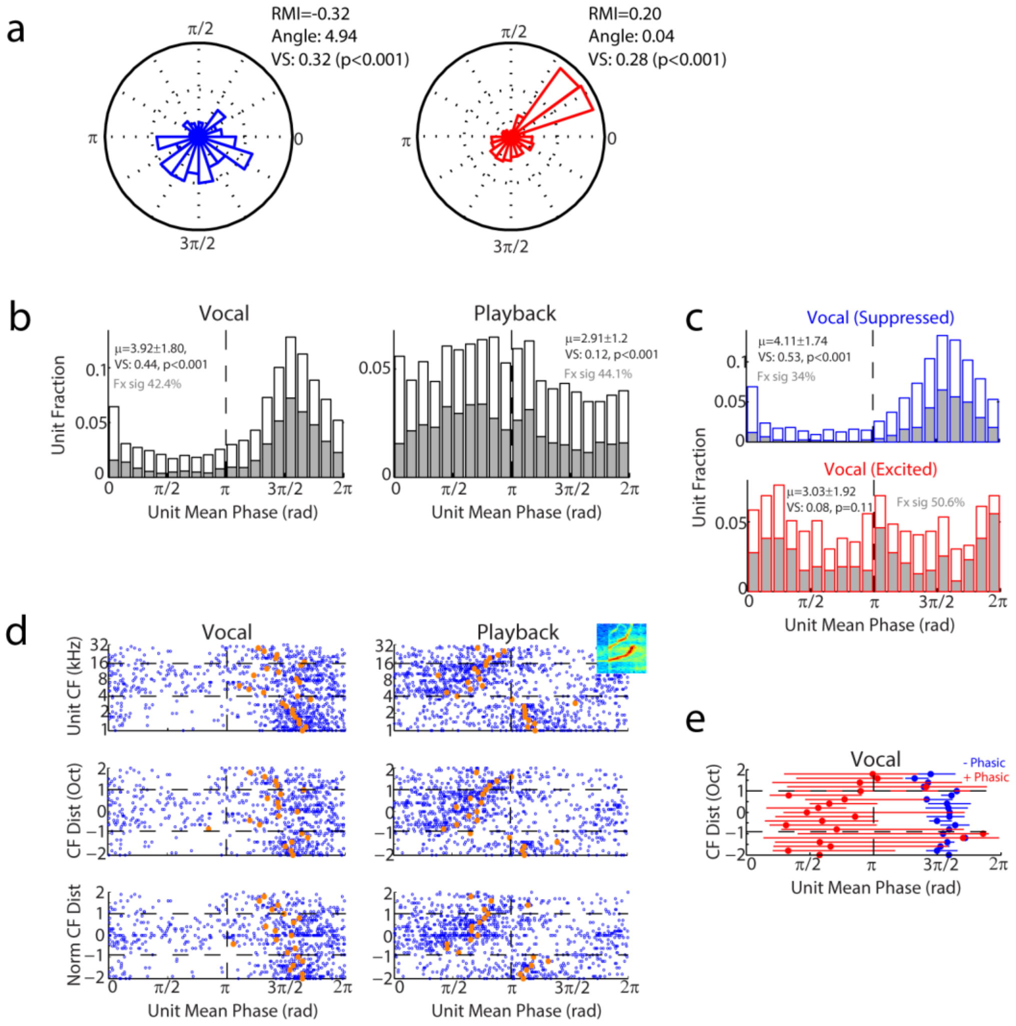

Supplementary Fig. 6: Comparison of spike timing between vocal production and playback.
a Sample circular distributions are shown for spike phase relative to twitter phrase-interval cycles for two example units, one suppressed (left) and one excited (right). Phases of 0 and 2π indicate the start of a twitter phrase and the next, with lower phases indicating spikes during a phrase, and higher values spikes during inter-phrase intervals. Vocal RMI, mean phase angle, and vector strength (VS) are indicated for each unit (left: n=118 spikes, $p=5x10^{-6}$; right: n=492 spikes, $p=8x10^{-19}$, p-values estimated from VS and Raleigh statistics). **b** Population distributions of mean phase angles for all units measured during vocal production (left), and playback (right). Shaded bins indicate units with significant vector strength (p<0.05), and the fraction of significant units is indicated (Fx sig). Population average vector strengths and p-values are indicated (vocal: $p<1x10^{-200}$, playback: $p=4x10^{-22}$, n=3248 units, VS and Raleigh statistics). During vocal production, there was a bias towards spikes in later phases (intervals), while playback was biased towards earlier spikes during phrases. **c** Comparison of population vocalspike phase distributions measured for suppressed and excited units. Excited units had more even distributions of their spike times than for suppressed units (suppressed: n=1508 units, $p=4x10^{-197}$; excited: n=393 units, p=0.11). **d** Unit spike phase means are shown compared to frequency tuning for vocal production (left) and playback (right). Results are shown separately for raw unit CF (top), CF distance from reference (middle), and distance normalized by bandwidth (bottom). Orange symbols indicate mean phase for a given frequency bin, and show a temporal pattern within vocal frequency ranges (dashed) for playback that is similar to the spectro-temporal pattern of twitter phrases (spectrogram inset), with earlier responses in lower frequencies compared to higher. **e** Mean spike phase during vocalization compared to CF distance for units with positive (red) and negative (blue) phasic vocal RMIs. Positive phasic units showed spikes earlier than negative phasic, and exhibit some of the temporal dynamics seen during playback. Mean and SEM are plotted (+phasic n=471 units, -phasic n=382 units). Source data are provided as a Source Data file.

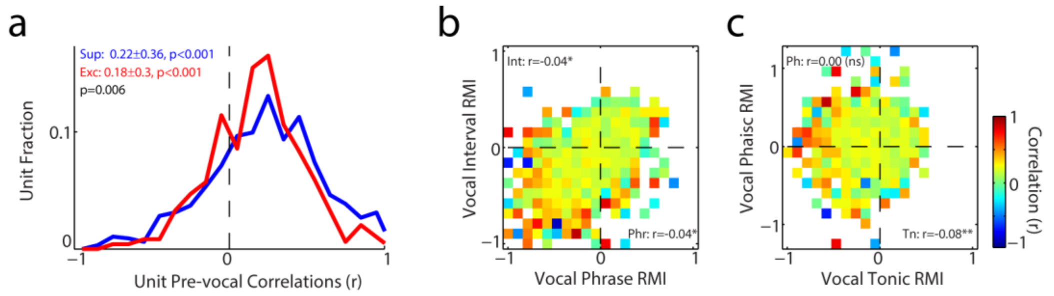

Supplementary Fig. 7: Comparison of pre-vocal correlations between suppressed and excited units.
a Distribution of correlation coefficients between vocal and pre-vocal activity calculated for individual units (as in Fig. 4c), divided into suppressed (blue, RMI≤-0.2) and excited (red, RMI≥0.1) populations (mean±s.d. indicated; sup: n=673 units, $p=8x10^{-44}$; exc: n=490 units, $p=3x10^{-32}$; two-sided signed-rank test). Suppressed units showed slightly stronger pre-vocal correlations than excited units (p=0.006, two-sided rank-sum test). **b,c** Unit correlation coefficients sorted by twitter phrase/interval (**b**) and phasic/tonic responses (**c**). Significant inverse correlations were noted to tonic/average responses, and to a lesser extent phrases and intervals, but not to P-I differences, consistent with stronger unit-level pre-vocal correlations in tonic, but not phasic, suppressed units (* p<0.05, ** p<0.001; Phr: p=0.038, Int: p=0.037, Tn: p=3x10-5, Ph: p=0.96; partial correlations and two-sided t-tests, n=2489 units). Source data are provided as a Source Data file.

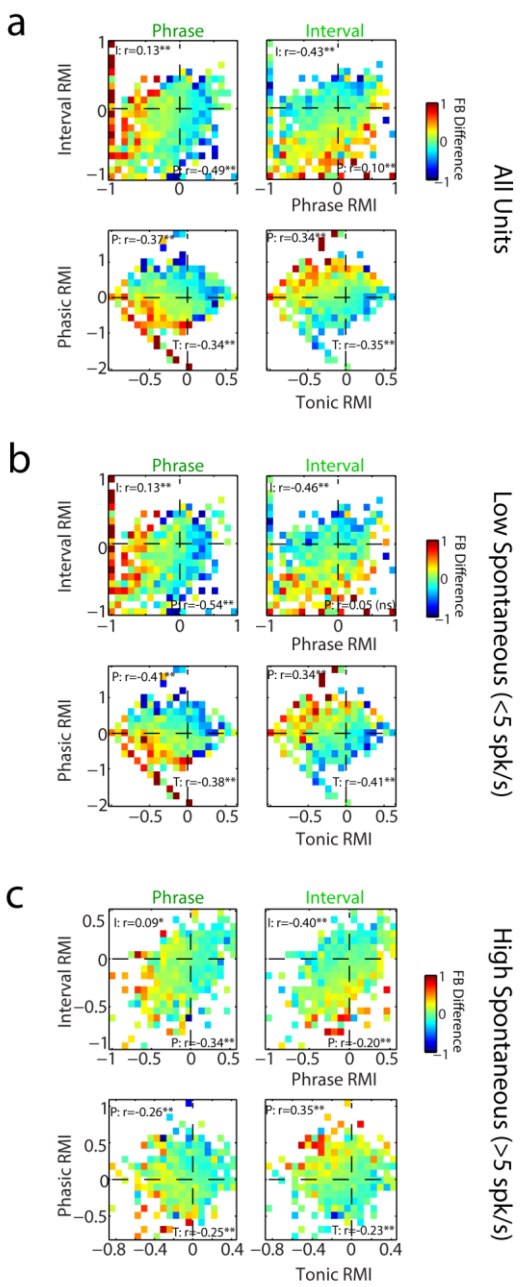

Supplementary Fig. 8: Effects of spontaneous firing rate on feedback responses.

a Feedback differences (feedback-normal) for all units, shown separately for feedback effects during phrases (left) and intervals (right). Results are shown separately sorted by normal phrase/interval (top; P: phrase, I: interval), and phasic/tonic response (bottom; P: phasic, T: tonic). Partial correlations are indicated (*p<0.05, **p<0.001; Phrase: P: p=7x10^{-142}, I: p=6x10^{-11}, T: p=1x10^{-63}, P: 1x10^{-78}; Interval P 4x10^{-7}, I 2x10^{-109}, T 1x10^{-68}, P 8x1$10^{-65}$; partial correlations with two-sided t-tests, n=2366 units). **b,c** Feedback effects in low-spontaneous rate (<5 spikes/sec, **b**) and high-spontaneous rate (>5 spikes/sec, **c**) units. Qualitatively similar correlations were noted between feedback effects regardless of spontaneous rate. Absolute magnitudes of feedback differences were smaller for high spontaneous units due to RMI normalization (see Methods). The absence of qualitative changes suggests feedback correlation with P-I differences were not simply due to biases introduced by a zero-firing rate floor during normal baseline vocalizations. (**B**: Phrase p-values: P 1x10^{-85}, I 1x10^{-5}, T 1x10^{-47}, P 2x10^{-46}; Interval: P 0.09, 1x10^{-59}, 2x10^{-45}, 1x10^{-3}; n=1099 units; **C**: Phrase: P 1x10^{-36}, I 8x10^{-4}, T 4x10^{-19}, P 7x10^{-21}; Interval: P x10^{-13}, I 3x10^{-50}, T 2x10^{-16}, P 2x10^{-38}; n=1267 units). Source data are provided as a Source Data file.
