# Supplementary material for: Multiple processes of vocal sensory-motor interaction in primate auditory cortex
Source: Nat Commun. 2024 Apr 10;15:3093. doi: 10.1038/s41467-024-47510-2 (PMC11006904; doi:10.1038/s41467-024-47510-2)
Supplement: Supplementary file 3 — Reporting Summary [file 41467_2024_47510_MOESM3_ESM.pdf]

nature portfolio

Corresponding author(s): Steven Eliades

Last updated by author(s): Feb 25, 2024

Reporting Summary

Nature Portfolio wishes to improve the reproducibility of the work that we publish. This form provides structure for consistency and transparency in reporting. For further information on Nature Portfolio policies, see our Editorial Policies and the Editorial Policy Checklist.

Statistics

For all statistical analyses, confirm that the following items are present in the figure legend, table legend, main text, or Methods section.

n/a	Confirmed	
☐	☒	The exact sample size (*n*) for each experimental group/condition, given as a discrete number and unit of measurement
☐	☒	A statement on whether measurements were taken from distinct samples or whether the same sample was measured repeatedly
☐	☒	The statistical test(s) used AND whether they are one- or two-sided<br>*Only common tests should be described solely by name; describe more complex techniques in the Methods section.*
☐	☒	A description of all covariates tested
☐	☒	A description of any assumptions or corrections, such as tests of normality and adjustment for multiple comparisons
☐	☒	A full description of the statistical parameters including central tendency (e.g. means) or other basic estimates (e.g. regression coefficient) AND variation (e.g. standard deviation) or associated estimates of uncertainty (e.g. confidence intervals)
☐	☒	For null hypothesis testing, the test statistic (e.g. *F*, *t*, *r*) with confidence intervals, effect sizes, degrees of freedom and *P* value noted<br>*Give P values as exact values whenever suitable.*
☒	☐	For Bayesian analysis, information on the choice of priors and Markov chain Monte Carlo settings
☒	☐	For hierarchical and complex designs, identification of the appropriate level for tests and full reporting of outcomes
☐	☒	Estimates of effect sizes (e.g. Cohen's *d*, Pearson's *r*), indicating how they were calculated

Our web collection on statistics for biologists contains articles on many of the points above.

Software and code

Policy information about availability of computer code

Data collection	Data were collected using software provided by Tucker Davis Technologies (hardware manufacturer) and custom code in Matlab (R2013)
Data analysis	Data were analyzed using custom code in Matlab (R2014). Code available from the corresponding author, without restrictions, upon request.

For manuscripts utilizing custom algorithms or software that are central to the research but not yet described in published literature, software must be made available to editors and reviewers. We strongly encourage code deposition in a community repository (e.g. GitHub). See the Nature Portfolio guidelines for submitting code & software for further information.

Data

Policy information about availability of data

All manuscripts must include a data availability statement. This statement should provide the following information, where applicable:

- Accession codes, unique identifiers, or web links for publicly available datasets
- A description of any restrictions on data availability
- For clinical datasets or third party data, please ensure that the statement adheres to our policy

Source data necessary to evaluate these results are provided. The raw datasets generated during and/or analyzed during the current study are available from the corresponding author on reasonable request. The size of the raw data sets exceeds that limitations imposed by current available repositories.

Research involving human participants, their data, or biological material

Policy information about studies with human participants or human data. See also policy information about sex, gender (identity/presentation), and sexual orientation and race, ethnicity and racism.

Reporting on sex and gender	This work did not involve human participants, data, or biological materials
Reporting on race, ethnicity, or other socially relevant groupings	This work did not involve human participants, data, or biological materials
Population characteristics	This work did not involve human participants, data, or biological materials
Recruitment	This work did not involve human participants, data, or biological materials
Ethics oversight	This work did not involve human participants, data, or biological materials

Note that full information on the approval of the study protocol must also be provided in the manuscript.

Field-specific reporting

Please select the one below that is the best fit for your research. If you are not sure, read the appropriate sections before making your selection.

☒ Life sciences ☐ Behavioural & social sciences ☐ Ecological, evolutionary & environmental sciences

For a reference copy of the document with all sections, see nature.com/documents/nr-reporting-summary-flat.pdf

Life sciences study design

All studies must disclose on these points even when the disclosure is negative.

Sample size	No sample size calculations were performed. All data collected were included, with the numbers of vocalizations and neurons recorded determined after conclusion of experiments. All neurons during which the appropriate type of vocalization (twitters) were included. No pre-determined samples size was chosen due to the unpredictibility of behavior and neural yields.
Data exclusions	No data were excluded from the overall results.. All animals and neurons with data from twitter vocalizations were included.
Replication	We collected the maximum number of vocalizations within the allowable experimental time, and their responses, from each recording site or neuron (typically between 4 and 100 twitters per session, but variable from day to day due to natural behavioral variability). Experiments were repeated over a number of different, independent experimental sessions (328 in total), with variable numbers of neurons recorded per session (3284 total), and for multiple separate animals (5 animals). These comprised all the experimental sessions during which twitter vocalizations were made by the animals, and no sessions or units were excluded.
Randomization	Not relevant to study. Timing of feedback or playback events were randomly decided by computer during vocal production. Only five animals were used, precluding randomization. All neurons recorded were studied, but some sessions used one feedback shift or the other, based upon order of recording session on a rotating basis, but without relationship to specific neural tuning or behaviors. Additional randomness introduced by animals' natural vocal behavior.
Blinding	Events were randomly triggered by computer, with no investigator involved during recording. During analysis, the reviewer was blinded to all feedback timing where possible.

Reporting for specific materials, systems and methods

We require information from authors about some types of materials, experimental systems and methods used in many studies. Here, indicate whether each material, system or method listed is relevant to your study. If you are not sure if a list item applies to your research, read the appropriate section before selecting a response.

Materials & experimental systems

n/a	Involved in the study
☒	☐ Antibodies
☒	☐ Eukaryotic cell lines
☒	☐ Palaeontology and archaeology
☐	☒ Animals and other organisms
☒	☐ Clinical data
☒	☐ Dual use research of concern
☒	☐ Plants

Methods

n/a	Involved in the study
☒	☐ ChIP-seq
☒	☐ Flow cytometry
☒	☐ MRI-based neuroimaging

Animals and other research organisms

Policy information about studies involving animals; ARRIVE guidelines recommended for reporting animal research, and Sex and Gender in Research

Laboratory animals	Five adult common marmosets (Callithrixx jacchus, both sexes, all >18 months old)
Wild animals	This study did not involve wild animals
Reporting on sex	Data were collected from 5 marmosets (4 male, 1 female). Animals were chosen based upon their willingness to vocalize under experimental conditions, and constrained by available animals in our breeding colony. Due to the small number of animals involved, typical of non-human primate research, no sex-specific analyses were performed. However, we observe broadly similar types of neural responses for animals of both sexes.
Field-collected samples	This study did not involve field-collected samples
Ethics oversight	All studies were performed under the guidance and protocols of the IACUCs at Johns Hopkins University (Johns Hopkins University Animal Care and Use Committee) and the University of Pennsylvania (University of Pennsylvania Animal Care and Use Committee).

Note that full information on the approval of the study protocol must also be provided in the manuscript.

Plants

Seed stocks	No plants were used in these experiments
Novel plant genotypes	No plants were used in these experiments
Authentication	No plants were used in these experiments
